# Community‐based dementia care models in India: A scoping review

**DOI:** 10.1002/alz70858_099759

**Published:** 2025-12-25

**Authors:** Vishnu Mangalamchery, Nalakath A. Uvais

**Affiliations:** ^1^ IQRAA International Hospital and Research Centre., Calicut, Kerala, India; ^2^ Iqraa International Hospital and Research Centre, Calicut, Kerala, India

## Abstract

**Background:**

Dementia is a significant public health issue in India, primarily affecting the aging population. Currently, 24.3 million people worldwide live with dementia, with 4.6 million new cases annually. This number is projected to double every 20 years, potentially reaching 81.1 million by 2040. In India, cases are expected to increase by 300%, highlighting an urgent need for effective community‐based care models. This scoping review aims to examine existing community‐based dementia care models in India and identify strategies to enhance support for individuals with dementia and their caregivers.

**Methods:**

A comprehensive search was conducted in PubMed, Google Scholar, and Scopus using predefined keywords such as “dementia care models,” “Alzheimer's care,” “community mental health,” “community psychiatry,” “daycare services for dementia,” “task shifting,” and “family caregiver support,” combined with “India.” The search included studies published in English without date restrictions. A total of 12 original research studies focusing on community‐based dementia care models in India were reviewed. Inclusion criteria encompassed articles detailing frameworks, interventions, and strategies tailored to the Indian context. Data extraction followed predefined guidelines, with two independent reviewers resolving discrepancies through discussion. Key aspects of community‐based dementia care models were analyzed to identify effective approaches and gaps in current practices.

**Results:**

Community‐based dementia care models in India encompass diverse approaches aimed at reducing the treatment gap and improving accessibility. Key strategies include the use of digital health technologies for late‐stage dementia management, task‐sharing through mobile technology, and tele‐mentoring of primary care physicians in dementia care. Additionally, community‐based memory clinics, outpatient and home‐based care integrated into community psychiatry services, and daycare centers for dementia patients have been implemented. Specialized dementia care hospitals and home‐based caregiver support programs were also identified. Integration of dementia care into primary healthcare settings was suggested as a scalable and sustainable model to address the growing needs of individuals with dementia and their caregivers.

**Conclusion:**

Expanding community‐based dementia care in India necessitates a collaborative, multi‐sectoral approach supported by robust policies. By prioritizing the development of sustainable care models, we can enhance early diagnosis, ensure appropriate treatment, and improve the quality of life for individuals with dementia.

